# Supplementary material for: Electrothermally Self-Healing Delamination Cracks in Carbon/Epoxy Composites Using Sandwich and Tough Carbon Nanotube/Copolymer Interleaves
Source: Polymers (Basel). 2022 Oct 14;14(20):4313. doi: 10.3390/polym14204313 (PMC9611350; doi:10.3390/polym14204313)
Supplement: Supplementary file 1 [file polymers-14-04313-s001.zip › polymers-1955605-supplementary.pdf]

Electrothermally Self-healing Delamination Cracks in Carbon/Epoxy Composites Using Sandwich and Tough Carbon nanotube/Copolymer Interleaves

Qin OuYang [1], Ling Liu [1,*] and Zhanjun Wu [2]

[1] School of Aerospace Engineering & Applied Mechanics, Tongji University, Shanghai 200092, China

[2] School of Aeronautics and Astronautics, Dalian University of Technology, Dalian 116024, China

* Correspondence: lingliu@tongji.edu.cn

Section S1. Reason of acid treatment

The presence of dispersant will reduce the electrical conductivity and electrothermal properties of the interleaf. The main component of the dispersant in this work is a modified polyvinyl alcohol, which can react with inorganic acids. According to ref. [1], after the CNT layer is treated with nitric acid for about 1 min, the reactants can be washed away by deionized water. Based on this, a control test was done and the results are shown in Figure S1 below. After treating the CNT-EMAA-CNT membrane or CNT/EP-EMAA-CNT/EP interleaf with nitric acid, the resistivity decreases, because polymer dispersant is removed and the interfacial resistance is eliminated to some degree. When the acid treating time reaches 60 or 75 s, the resistivity changes slowly or nearly steadily, indicating that the acid treating time is already suitable. Therefore, CNT/EMAA with acid treatment for 75 s is used in this work.

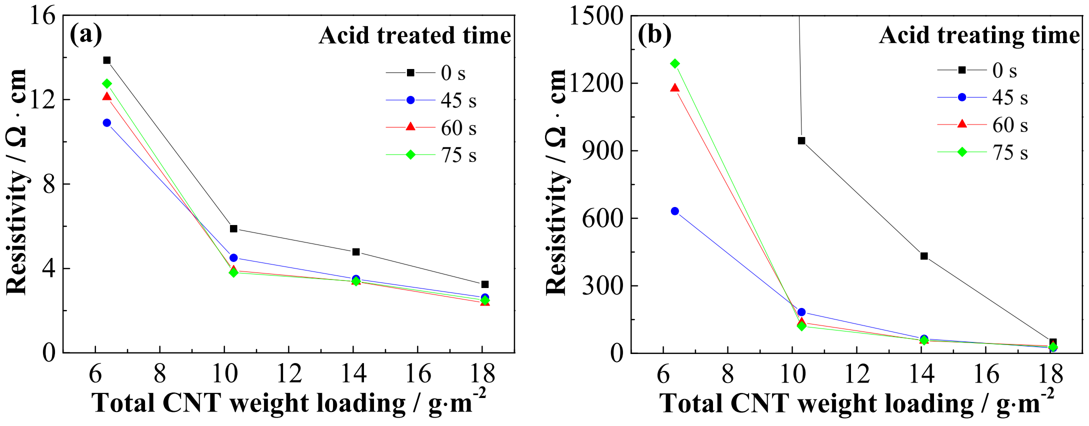

Figure S1. **(a)** Resistivity of the CNT-EMAA-CNT membrane, **(b)** resistivity of the CNT/EP-EMAA-

CNT/EP interleaf.

Section S2. Preparation of the CNT/EP/EMAA "off-line" interleaves

Firstly, the CNT-EMAA-CNT, EMAA-CNT and EMAA membranes were cut into rectangle with dimension of 50 mm×50 mm. Then, the CNT-EMAA-CNT was directly coated with an EP/DMDC mixture, where the weight ratio of EP to DMDC is 100:35 and followed by putting into a mould cavity and sealed with a vacuum bag (named as CNT/EP-EMAA-CNT/EP). As for the EMAA-CNT, EP is coated on the surface of CNTs to form CNT/EP layer and then another EMAA film is laid on the CNT/EP, thus this interleaf is named as EMAA-CNT/EP-EMAA. During the curing process, the temperature was firstly raised from room temperature to 60 °C and held for 4 h, then raised to 150 °C and kept for 2 h.

Section S3. Characterization of Resistivity and electrical-heating rate

The CNT/EP/EMAA specimens were used to measure the resistivity. Before testing, both ends of the specimens were polished and coated with conductive silver adhesive and then bonded with copper foil to form testing electrodes. A Keithley 2450 current source meter was used to measure the electrical resistances using two-point method. The above specimens were also electrified in direct-current mode with various voltage. During the electrical heating process, some typical temperature fields of the specimens were recorded by a FLIR ONE infrared camera.

Reference

1. Gao, Y.; Liu, L.; Wu, Z.; Zhong, Z. Toughening and self-healing fiber-reinforced polymer composites using carbon nanotube modified poly (ethylene-co-methacrylic acid) sandwich membrane. *Compos. Part A Appl. Sci. Manuf.* **2019**, *124*, 105510.
